# Influence of Electrode Polishing Protocols, Potentiostat Models, and LOD Calculation Methods on the Electroanalytical Performance of SWV Measurements at Glassy Carbon Electrodes

**DOI:** 10.3390/molecules30234651

**Published:** 2025-12-04

**Authors:** Michał Świderski, Jagoda Seroka, Dariusz Guziejewski, Paweł Krzymiński, Alicja Miniak-Górecka, Kamila Koszelska, Nabi Ullah, Sylwia Smarzewska

**Affiliations:** 1 Doctoral School of Exact and Natural Sciences, University of Lodz, 90-237 Lodz, Poland; 2 Department of Inorganic and Analytical Chemistry, Faculty of Chemistry, University of Lodz, 91-403 Lodz, Poland; 3 Department of Intelligent Systems, Faculty of Physics and Applied Informatics, University of Lodz, 90-236 Lodz, Poland

**Keywords:** polishing, electroactive surface, voltammetry, glassy carbon electrode, potentiostat, limit of detection

## Abstract

The aim of this research is to present the extent to which the basic elements used in electrochemical measurements affect the results of electroanalytical procedures. Measurements were carried out using the square wave voltammetric technique on a glassy carbon electrode, and the recorded analytical signal corresponded to a model redox system. One of the objectives of the study was to illustrate the impact of using potentiostats from different manufacturers, as well as the variations observed among different models within the same brand. These models exhibited notable differences in both cost and advanced electrochemical measurement capabilities. The mechanical cleaning method for the solid disk electrode surface was also taken into consideration. Three different polishing motion types were tested, together with the number of repetitions. It was revealed that polishing motion significantly influences the electroactive surface area of the working electrode, as well as the repeatability of the measurements. The research showed that the largest electroactive surface area and the best repeatability of parameters are achieved when polishing is performed by drawing an 8-type motion on the polishing pad. The obtained results confirmed that the equipment and polishing applied in research have a greater than previously assumed impact on the statistical parameters characterizing the analytical procedure, for example, the limit of detection (LOD) or the dynamic range of the calibration curve. Both analyzed parameters have a significant impact on the quality of the statistical parameters describing derived analytical procedures. Finally, it was shown that significantly different statistical parameters can be obtained from the same set of data using various approaches for LOD estimation, with discrepancies reaching up to two orders of magnitude.

## 1. Introduction

Electrochemistry is a specialized field of chemistry that focuses on processes involving electron exchange with an external source. It covers the study of electrolytes, electrical conductivity, and electrode reactions, with applications in analytical chemistry, biology, medicine, renewable energy, and environmental protection [1,2,3]. Electrochemical studies delve into understanding mechanisms involving accompanying chemical reactions, adsorption processes, and other related phenomena [3,4].

Voltammetric measurements are most often made using a three-electrode system, which includes the working, reference, and auxiliary electrodes. The electrochemical processes of interest that are the basis of the analysis take place at the working electrode (WE) surface or in its close vicinity. Depending on the applied potential, this electrode can function as a cathode (−) or anode (+), i.e., it is polarizable and can serve as a source of electrons during reduction or a sink during oxidation processes.

Mercury electrodes were once widely used in electrochemical measurements due to their excellent reproducibility and easily renewable surface [5]. However, because of the volatility and toxicity of mercury and the related environmental and health risks, their use has significantly declined. They were replaced by safer solid electrodes made of carbon materials (e.g., glassy carbon, pyrolytic graphite, boron-doped diamond) [6,7,8,9] or precious metals (e.g., platinum, gold, silver) [10,11] and later by electrodes modified with various organic or inorganic materials [12,13,14,15].

Currently, solid-phase working electrodes dominate, and analytical results depend strongly on their material, geometry, and surface morphology. The electrode material should ensure high electrical conductivity, a wide potential window, and a favorable signal-to-noise ratio, while remaining chemically inert, mechanically stable, and cost-effective [16,17].

Among them, glassy carbon (GC) is the most commonly used. It is an amorphous, non-graphitizing carbon formed by pyrolysis above 2000 °C [18], consisting of defective polycrystalline domains with randomly distributed graphene-like regions [19,20]. Glassy carbon electrodes (GCEs) combine hardness, chemical and thermal stability, low impedance, and reusability due to the ease of surface renewal [21]. Compared to graphite, GC exhibits greater hardness but slightly lower electrical conductivity [6,21].

GCEs exhibit a constant surface area, but during measurements, they can become contaminated and affected by the applied potential. Therefore, the working electrode needs to be refreshed before subsequent measurement. Three methods of electrode surface renewal are used most frequently: mechanical, chemical, and electrochemical. Chemical cleaning involves washing the electrode with acids (e.g., sulfuric acid (VI) or nitric acid (V)), bases (sodium or potassium hydroxide), organic solvents (e.g., isopropyl alcohol or acetone), or electrolyte solutions that dissolve surface deposits [22,23,24]. Electrochemical cleaning relies on potential cycling (typically at 0.1 V s^−1^) or applying highly positive or negative potentials to remove contaminants through oxidation or reduction reactions [25]. The electrochemical cleaning process may vary depending on the type of electrode used and the bond character of an attached contaminant. For metal electrodes (e.g., Pt, Au), such regeneration dissolves impurities. For carbon electrodes, various electrochemical purification methods can also be used, for example, electrolysis in acidic or basic solutions [23]. However, the most common approach is mechanical cleaning using abrasive materials such as sandpaper, brushes, or polishing pastes. Polishing with a pad and abrasive suspension is preferred [24], typically employing particles decreasing from about 30 μm to 0.05 μm [26], applied on appropriate polishing pads [27], and performed with selected polishing motions.

The selection of an appropriate working electrode is crucial for optimizing any electroanalytical procedure, while its maintenance and cleaning are essential for ensuring data quality. However, the choice of a potentiostat is also an important aspect, often overlooked and typically dictated by the equipment available in a given laboratory. A potentiostat is an electronic device used in voltammetric experiments. Its operation involves applying a specified potential to the working electrode relative to the reference electrode potential using a high-resistance measurement circuit (e.g., 10 MΩ) to eliminate current flow through the reference electrode. This instrument consists of many internal components with diverse functionalities designed to control and measure electric parameters such as current, potential, and resistance (like a control amplifier, current and voltage followers, signal generator, etc.). Most available potentiostats can also operate in galvanostatic mode, i.e., controlling current flow rather than potential. Another optional feature is the ability to function as a bipotentiostat, i.e., to control two working electrodes at once. All these characteristics enable work with digital control over two-, three-, and even four-electrode systems for any electrochemical technique available [3], and even to apply, for example, user-defined potential alteration programs [28,29,30].

Various potentiostat models differ significantly in their parameters, which should be considered during the planning and selection stages of research. Compact potentiostats enable *on-site* or remote analyses, even via smartphone applications and Bluetooth connectivity, offering excellent potential for environmental studies [31]. In contrast, larger modular laboratory devices, though less portable, provide advanced functionality and allow for greater flexibility and precision in electrochemical measurements [28].

Evaluation of any analytical approach for analyte determination ultimately requires statistical analysis that confirms the quality of the analysis. Parameters such as repeatability, precision, recovery, and accuracy are commonly evaluated. Validation of the developed methodology also defines the dynamic linear range, as well as the limits of detection (LOD) and quantification (LOQ). The limit of detection is the lowest signal, or the lowest corresponding quantity to be determined from the signal, that can be observed with a sufficient degree of confidence or statistical significance. Although its definition is quite simple and clear, the methods of its calculation can differ substantially depending on the chosen approach [32,33]. As a result, the resulting threshold values may vary widely, influencing the overall assessment of the analytical method’s quality.

In this study, we aimed to comprehensively investigate the influence of fundamental experimental variables on electroanalytical measurements. Specifically, we examined the impact of key elements such as the type of potentiostat device, mechanical polishing parameters (including polishing motion type and number of movements), and the method of determining the limit of detection. All experiments were performed using square wave voltammetry on a glassy carbon electrode, and we analyzed the resulting calibration curves for a model redox couple. Our findings provide valuable insights into how these basic elements affect the accuracy, precision, and sensitivity of electroanalytical techniques. This research contributes to a deeper understanding of the factors influencing electrochemical measurements and provides the groundwork for optimizing experimental protocols to enhance analytical performance.

## 2. Results and Discussion

### 2.1. Influence of Polishing Motion Type

There are different types of WEs: liquid (mercury drop electrodes, e.g., HMDE or DME), paste, and solid electrodes (carbon, metallic, etc.) in the form of a disk. The choice of the appropriate working electrode depends primarily on the value of the redox potential of the tested compound. It should also be kept in mind that working electrodes have different polarization ranges depending on the pH of the environment. Currently, solid electrodes are used most often, as they are safer than mercury electrodes; however, unlike the latter, in which the geometric surface area of the drop is equal to the real one, the surfaces of solid electrodes are not uniform. Therefore, at different places on the surface, electrode processes occur with non-uniform electron transfer rates, and such electrodes or processes cannot be characterized easily [34].

WEs used in electroanalytical studies become contaminated with analytes or electrode reaction products [35]. That may reduce sensitivity and repeatability but also shorten the electrode lifetime. On the WE surface, adsorption of poorly soluble compounds, passivation, electrode corrosion, or degradation due to mechanical or chemical damage (such as radiation, reaction with the electrolyte, electrode aging, etc.) may occur. The response of the system, involving the kinetics of charge transfer between the analyte and the electrode, depends on the condition of the working electrode surface; therefore, it is crucial to keep it as clean and well-defined as possible. Surface treatment may result in prolonging the duration of the studies and introducing additional chemical reagents and instrumental methods, which increases the research complexity and time; however, it is absolutely necessary. Depending on the type of electrode, different refreshment methods can be used. As already mentioned in Section 1, there are three types of methods for regenerating the surface of solid electrodes: mechanical, chemical, and electrochemical. The most frequently used method, and the subject of this research, is the mechanical cleaning method. It should also be mentioned that the WE surface after polishing is not perfectly clean, and the abraded particles may get stuck in the pores of the electrode; for this reason, it is most reasonable to additionally apply an ultrasonic bath. During ultrasonic cleaning, the electrode is immersed in an appropriate solution, in which pressure waves are created as a result of the cavitation phenomenon [36]. Before using this procedure, it is necessary to confirm with the electrode manufacturer whether the procedure will not damage its interior parts (e.g., may cause cracking due to conductive adhesives susceptible to such processes).

**Figure 1:**
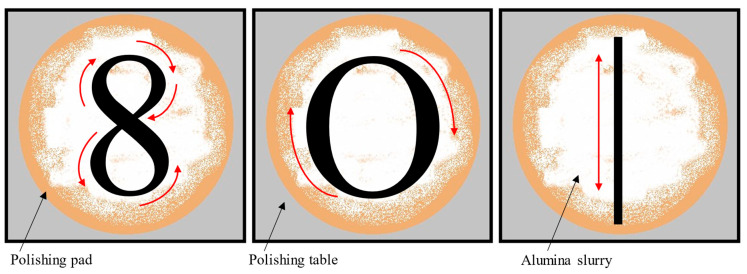
Polishing motion types: 8-type (**left**), O-type (**center**), I-type (**right**).

In this research, three polishing motion types were tested: 8-, O-, and I-type (Figure 1). The working electrode was refreshed mechanically (using the chosen polishing motion type) on a polishing cloth moistened with deionized water, along with an aluminum oxide suspension.

**Figure 2:**
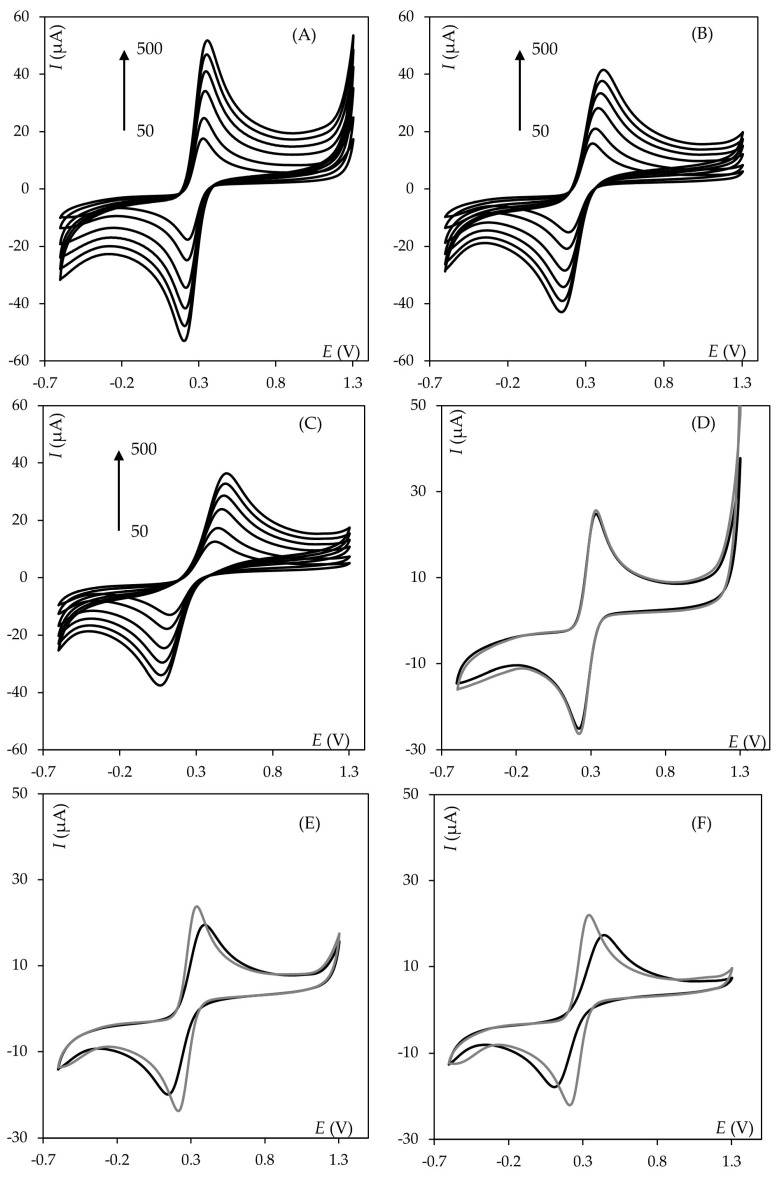
Cyclic voltammograms recorded for equimolar hexacyanoferrate(II)/hexacyanoferrate(III) mixture c = 1 × 10^−6^ mol mL^−1^ using different scan rates from the range 50–500 mV s^−1^ after application of polishing (**A**) 8-, (**B**) O- and (**C**) I-type; cyclic voltammograms showing the two most different measurements recorded at a scan rate of 100 mV s^−1^ after application of (**D**) 8-, (**E**) O-, and (**F**) I-type polishing motion.

For each type of polishing motion, nine series were conducted, with the results averaged after every three (subseries). During the 8-type polishing method, the time taken to complete thirty motions on the polishing cloth was measured, and then the average time value for all nine series was calculated. For the other two polishing methods, “circles” and “lines” were drawn for exactly the same time on the polishing cloth. After each refreshment, cyclic voltammograms of a 1 µmol mL^−1^ hexacyanoferrate mixture for different scan rates (50–500 mV s^−1^) were recorded in the potential window from −0.6 to 1.3 V (cf. Figure 2) with NaCl as the supporting electrolyte. Subsequently, using the modified Randles–Ševčík equation [37], electroactive surface area, Ã, was calculated (Table 1). In this evaluation, a 7.6 × 10^−6^ cm^2^ s^−1^ diffusion coefficient was assumed, and the anodic part of the voltammograms was taken into consideration.

As can be seen, 8-type polishing motion ensures excellent refreshment of the working electrode surface and, above all, very good repeatability, which is of great importance when performing electroanalytical measurements. For O- and I-type cleaning, exemplary voltammograms are noticeably not identical (cf. Figure 2E,F). Additionally, measurements at the same scan rate values are not perfectly convergent, and the peak currents are much lower than those obtained during 8-type polishing. What is more, WE electroactive surfaces are smaller and have worse repeatability. Apparently, the quality of electrode surface refreshment also affects anodic and cathodic peak potential separation, suggesting that electron transfer rates become more sluggish. Also, it can be observed that half-peak width increases in the sequence 8-, O-, and I-motion, confirming most probably the same effect. Comparing all polishing motions at the positive vertex potential, it can be observed that the highest increment in the current is for the 8-type motion, which could suggest that electrochemical activation is the most effective. WEs surfaces were examined with an optical microscope using 50× magnification, and no significant differences in surface morphology were observed. Next, the influence of the number of movements was studied for the case of the 8-type pattern. Here, apart from what was described above, we applied 10 and 50 repetitive movements. However, compared to the polishing method involving thirty 8-type movements, a smaller electroactive surface area was obtained, which was characterized by lower repeatability Ã = 0.10094 ± 0.00182 [cm^2^], CV = 3.86% for ten movements, Ã = 0.09927 ± 0.002958 [cm^2^], CV = 5.28% for fifty movements). The measured anodic currents for measurements recorded at the same scan rate values do not differ significantly. Although the obtained voltammograms are not identical, the scans overlaid at the same scan rate values practically coincide. However, most significant discrepancies emerge among the series labeled as “50”, particularly noticeable in the measured values of anodic peak currents. This observation contrasts with the results obtained from various numbers of 8-type motions during electrode polishing, suggesting a cumulative effect of increased motion count.

### 2.2. Influence of Apparatus

Potentiostats vary in the range or threshold values of many parameters characterizing their efficiency and quality, the most important of which include the maximum recorded current, the range of potentials applied, the current range, and number of current ranges as well as potential resolution. Because of the possible use of a potentiostat for electrochemical measurements with quite wide characteristics of the measured parameters, it is worth noting that the value of the maximum current determines the upper range of the current that can be measured by the device; therefore, it is not possible to measure a current higher than the maximum current value. Moreover, during research, the current range should be adjusted to the currently measured experimental values, which enables measurements without loss of precision. Manufacturers most often provide the lowest and highest available current range along with the number of decades. The use of an inappropriate range may result in high noise in the obtained response or a decrease in measurement accuracy. A simple solution in such a case is the option of automatic switching between the appropriate current range, adapted to the currently performed measurement. Another important feature of the potentiostat is its range of applied potentials, which determines the range of values that can be applied to the electrode surface. This parameter is also crucial due to the type of application area (corrosion tests, fuel cells, etc.), and in the case of redox reactions, which are the basis of electroanalysis, precise control of the range and value of the potential is extremely important. The last important parameter to consider is potential resolution, both applied and measured. Generally speaking, the higher the resolution, the greater the measurement accuracy.

In the second stage of the research, using optimized mechanical polishing, five different potentiostats (Table 2), manufactured by different companies and with different general parameters discussed in the introduction, were compared. The first one was PalmSens’ EmStat3 with PSTrace version 4.7. It is a compact, small-sized potentiostat, whose indisputable advantage is its relatively low price. It enables analysis based on numerous voltammetric techniques implemented in the software. Another potentiostat was the µStat 200 by DropSens with DropView 200 software. The µStat 200 is a small and portable potentiostat, similar in size to the previous one, but it also allows for measurements on two electrodes working simultaneously (so-called bipotentiostat). Similarly to the previously described instrument, it offers amperometric and voltammetric measurements (including a wide variety of voltammetric techniques). Three more potentiostats were manufactured by the same company, namely Autolab (formerly Eco-Chemie). The first one was the Autolab 302N, which is the highest quality potentiostat offered by this manufacturer. It is characterized by a high current range of up to 2 A (and even up to 20 A if an additional amplification module is used) and a maximum voltage between the working and auxiliary electrodes of up to 30 V. The device is controlled by modern NOVA software. Multi Autolab/M101 is another potentiostat, also controlled by NOVA software. It is a multi-channel potentiostat equipped with a number of modules based on M101 potentiostats, which means that it is possible to conduct measurements independently or in parallel for up to 12 subunits (or 6 if modules are combined with an additional hardware module). The last potentiostat used during the measurements was the µAutolab type III. It was one of the cost-effective options offered by Autolab (now replaced by the 101 model). When using this equipment, GPES software version 4.9 was used (predecessor to the currently offered NOVA control software from Autolab). Three were selected from the same manufacturer because, at the initial stage, the Emstat3, µStat 200, and µAutolab type III were examined, and the latter performed so well that two other significantly more expensive and advanced models were also tested. The main technical parameters of each of the potentiostats used are listed in Table 2. All parameters were taken from the user manuals of the compared instruments and are available on the manufacturers’ websites [38,39,40,41].

**Figure 3:**
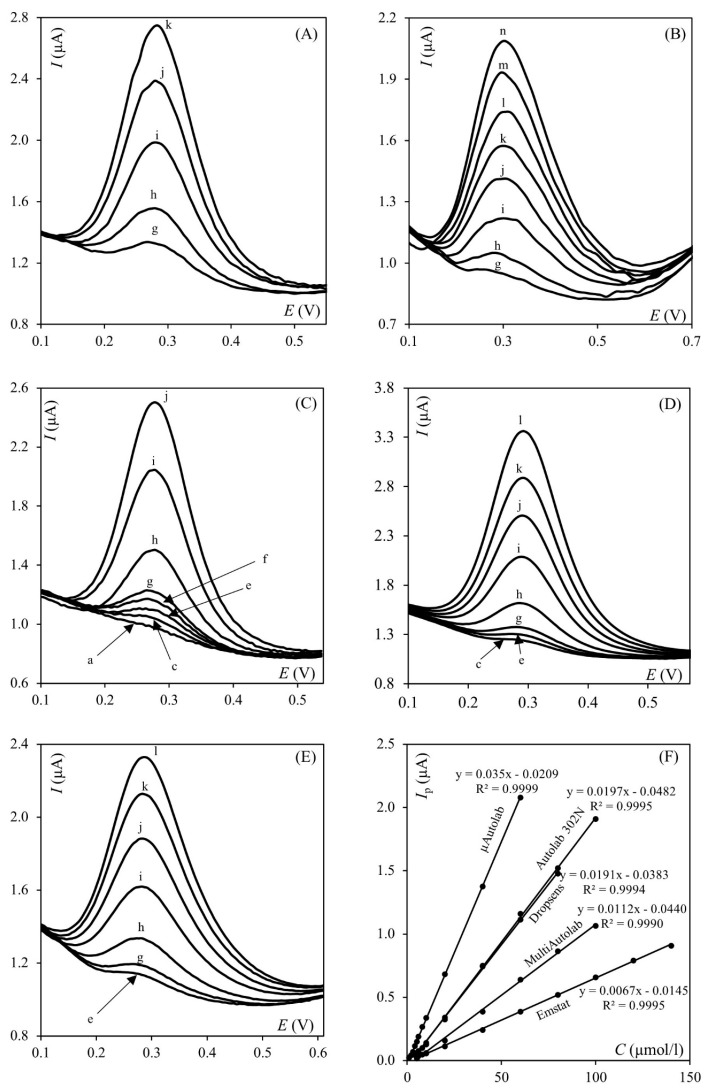
Square wave voltammograms ((**A**) µStat 200, (**B**) Emstat3, (**C**) µAutolab, (**D**) Autolab 302N, (**E**) Multi Autolab/M101) and corresponding calibration curves (**F**), potassium hexacyanoferrate (II/III) concentrations: (a) 2.0, (b) 3.0, (c) 4.0, (d) 5.0, (e) 6.0, (f) 8.0, (g) 10.0, (h) 20.0, (i) 40.0, (j) 60.0, (k) 80.0, (l) 100.0, (m) 120.0, (n) 140.0 µmol·L^−1^.

The determination of the potassium hexacyanoferrate (II/III) mixture was performed using the SWV technique and NaCl as the supporting electrolyte. The signal from analyte oxidation was observed at a potential of ca. 0.24 V for each of the potentiostats used in the experiment. Three measurement series were performed on each apparatus and used to construct a calibration curve and determine the dynamic linear range. The results obtained for each of the potentiostats used are presented in Table 3. Figure 3 represents the received calibration curve voltammograms recorded with each of the potentiostats used (A–E), as well as calibration curves and their equations (F).

The calibration curves were constructed for measurements in which the same concentration interval was considered (8.0 × 10^−7^–2.0 × 10^−4^). The only problem encountered was with µStat 200, where the controlling software was unable to detect signals both with automatic and manual selection, although they were clearly visible. Based on the presented data, it can be concluded that significant differences were observed in the obtained results depending on the type of potentiostat used. The widest linear range was observed for both the µAutolab type III and Autolab 302N, indicating that they produce qualitatively similar results. Conversely, the shortest linear range was observed when using µStat 200. As depicted in the results presented in Table 3, the most sensitive method in terms of detecting the lowest concentration within the dynamic linear range was obtained with the µAutolab type III. In contrast, using the EmStat3 and µStat 200, the lowest concentration within the linear range was as high as 1.0 × 10^−5^, which compromised the statistical parameters of both devices. Another important parameter to consider is the slope of the obtained calibration curves, which influences the sensitivity and robustness of the procedure. Generally speaking, the greater the slope, the higher the sensitivity and robustness. In this case, the highest slope was obtained with µAutolab type III, while the smallest was obtained with EmStat3. Thus, it can be tentatively stated that, based on the obtained data, the µAutolab type III assumes a leading position. However, it is worth noting that the limit of detection (LOD) calculated using four different methods does not necessarily support this conclusion. Conversely, while the results obtained with the EmStat were not as satisfactory, their statistical evaluation parameters do not confirm this assessment. The quality assessment of the results must necessarily include an analysis of all possible parameters rather than relying solely on the slope of the calibration curve or minimizing the limit of detection. Valuable quality assessment parameters may include precision, repeatability, accuracy, selectivity, and measurement stability. It is also necessary to compare these parameters for different measurement methods to obtain a fuller picture of their effectiveness and suitability for a specific application. Additionally, it is important to consider the research context and the requirements and limitations of the analyzed sample or measurement system when assessing the quality of the results.

### 2.3. Influence of LOD Calculation Method

As illustrated in Section 3.2, we have at our disposal a diverse range of equations tailored to specific requirements. In this study, four distinct methods for calculating LOD were employed. These methods encompass a variety of conditions and scenarios, providing flexibility and versatility. The obtained results unequivocally indicate that depending on the method used for calculations, the obtained values significantly differ from each other, and it is challenging to observe any correlation. Despite the fact that the lowest concentrations in the linear range were observed when using µAutolab type III, only in the case of method four, LOD^4^, was the lowest LOD value compared to other potentiostats obtained. Using the first method of calculation, LOD^1^, the lowest LOD value was obtained for Multi Autolab/M101. The second method allowed the obtaining of the lowest LOD value for Autolab 302N. And surprisingly, in the case of the third method, LOD^3^, the lowest LOD value was obtained for EmStat3.

In many textbooks, different methods are suggested for various conditions, allowing users to select the one that best suits their requirements. However, when it comes to comparing the limit of detection (LOD) or limit of quantification (LOQ) of different methods, the process can be confusing, particularly in electrochemistry. Many of these methods appear to be influenced by noise, as exemplified by the Emstat case, where noise levels are significant despite acceptable LOD values. An interesting point is that, depending on the equation or method used for LOD calculation, we can identify each time a different device with the lowest value. On the other hand, although each calculated LOD is significantly lower than the first concentration from the dynamic linear range, it does not help in improving the sensitivity of the method. Therefore, care must be taken when comparing literature values using only LOD comparisons without knowledge of the particular methodology applied. 

## 3. Experimental

### 3.1. Apparatus and Solutions

The following potentiostats were used during the research: µAutolab type III with GPES 4.9 software (Metrohm Autolab B.V., Utrecht, The Netherlands), Autolab PGSTAT302N and MultiAutolab/M101 with Nova 2.1 software (Metrohm Autolab B.V., Utrecht, The Netherlands), µStat 200 with DropView 200 software (Metrohm-DropSens, Oviedo, Spain), and EmStat3 with PSTrace 4.7 software (PalmSens, Houten, The Netherlands). The following parameters of the SWV technique were used during measurements: frequency 25 Hz, potential step 5 mV, and amplitude 25 mV. A classic three-electrode system was used: glassy carbon (φ = 3 mm, Mineral, Lomianki-Sadowa, Poland) as the working electrode, silver chloride Ag/AgCl (3 mol·L^−1^ KCl, Mineral) as the reference electrode, and a platinum wire (Mineral) as an auxiliary electrode. Before each measurement series, the working electrode surface was mechanically renewed by polishing it on a pad (73 mm diameter microcloth polishing pad, Buehler, Lake Bluff, IL, USA) with the addition of an Al_2_O_3_ suspension (ATM Qness GmbH, Mammelzen, Germany). A stock solution of potassium hexacyanoferrates (II/III) mixture (Merck, Darmstadt, Germany, purity ≥ 99.0%) with a concentration of 0.1 mol·L^−1^ each was prepared by dissolving an appropriate amount of the analytes in deionized water. This solution was then diluted to obtain appropriate working concentrations. Sodium chloride (99.9% from POCh, Gliwice, Poland) with a concentration of 0.1 mol·L^−1^ was used as the supporting electrolyte. All solutions were stored in a refrigerator between measurements. The surface of the working electrodes was characterized using a HUVITZ HR3-TRF microscope (Huvitz Co., Ltd., Anyang-si, Republic of Korea).

### 3.2. Equations

The electroactive area (*A*) of the working electrode was calculated based on the modified Randles–Ševčík formula, which, for a quasi-reversible process, is as follows:

$$\left|I_{p}\right|=k\cdot z^{\frac{3}{2}}\cdot A\cdot c\cdot D^{\frac{1}{2}}\cdot v^{\frac{1}{2}}\cdot K(\Lambda ,\alpha )$$

where *I*_p_ refers to the peak current [A], *k* is the Randles–Ševčík constant, equal to 2.69 × 10^5^ [C·mol·V^1/2^] for T = 298 K, *z* is the stoichiometric number of exchanged electrons, *A* is the electroactive area [cm^2^], *D* is the diffusion coefficient of the chosen redox probe [cm^2^ s^−1^], *c* is the bulk concentration of redox probe [mol cm^−3^], *ν* is the potential scan rate (V s^−1^), and K(Λ,α) is a modified dimensionless parameter for quasi-reversible reactions [37].

The calibration curves (y = ax + b) were obtained by plotting the measured peak current (*I*_p_) against increasing redox probe concentration. Confidence interval (CI) was calculated as: CI = t(*SD*/n^1/2^), *p* = 95%, n = 3, where t is the t-value, *SD* is the sample standard deviation, and n is the sample size. Coefficient of variation (CV) was calculated using the following equation: CV = (*SD*/*ave*) × 100% where *ave* is the average of measured values.

In the present study, four different methods were used to evaluate LOD. All methods are described below:

First method, LOD^1^, is based on the signal-to-noise ratio (S/N), wherein the signal corresponding to the method’s detection limit should exhibit a minimum value that is at least three times higher than the noise signal (S/N ≥ 3) [32,33]. Here, the S/N = 3 was explicitly used.

The second method of estimating the LOD, LOD^2^, involved calculating this parameter based on the results obtained from blank sample measurements (the sampling point of the current located at the potential of the redox probe expected signal). More precisely, ten measurements were performed for ten independent blank solutions, then for the obtained voltammetric data, the average (*ave*) and standard deviation (*SD*) were calculated, and LOD values were estimated as: LOD = *ave* + 3*SD* [32].

In the next method, the limit of detection, LOD^3^, was calculated from the obtained calibration curves using the following equation: *k*·*SD_i_*/a, where *k* = 3, *SD_i_* is the standard deviation of the intercept, and *a* is the slope of the calibration curve [33].

The last procedure was also based on calibration curves; however, for LOD^4^, weas calculated as LOD = 3*SD*_Ip_*a*^−1^, where *a* has the same meaning as previously, and *SD*_Ip_ is the standard deviation of the obtained peak heights *I*_p_ (n runs for the lowest concentration observed in the calibration curve) [42].

## 4. Conclusions

Precision and repeatability are paramount concerns in the presentation of scientific results. Researchers must carefully consider various factors, including laboratory conditions and the purity and quality of the substances used. Our study underscores the importance of apparatus selection, electrode maintenance, and the equations governing statistical parameters that describe the quality of established procedures. These conclusions are based on experiments performed using square wave voltammetry on a glassy carbon electrode.

The first part of this research aimed to determine the optimal motion pattern (“eight”, “circle”, or “line”) to be drawn on a polishing cloth during the mechanical polishing of a glassy carbon working electrode surface. The most effective renewal of the electroactive surface of the working electrode was provided when the 8-type polishing motion was used. This type of motion resulted in nearly identical values of the electroactive surface area, ensuring reliable measurements characterized by high precision and repeatability. It was also confirmed that the number of repetitions performed during GCE polishing is equally important. The best results were obtained when the 8-type pattern was traced for 22 s (30 repetitive motions). Notably, any number of “eight” motions during polishing yielded better results than other motion patterns.

As polished glassy carbon is a part of a three-electrode system controlled by a potentiostat in the second stage, it was shown that different potentiostat models significantly influenced the obtained calibration curves, affecting both the dynamic range and the precision of the recorded concentration-dependent signal. During testing, the oxidation peak of the chosen model redox system appeared at the same potential vs. the reference electrode for each of the devices used in the experiment, indicating the uniform application of potential in all cases. The results showed smaller discrepancies between potentiostats from the same manufacturer than between those from different brands. This is most likely due to the use of similar technology and hardware in devices from one manufacturer. The variety of operating software could also have influenced the final results. An interesting example here is the µStat 200. During the measurements, its software automatically excluded “too small” peak currents from analysis in the recorded voltammograms, despite their clear visual presence.

In the final step, we calculated the limit of detection using various methods and revealed that comparing LODs from different analytical procedures, as often seen in review articles, may be unjustified if calculations were performed with different methods. Surprisingly, different potentiostats yielded the lowest LOD depending on the method used.

When initiating this study, the authors anticipated observing only minor differences. However, the obtained results were substantial enough to suggest that other researchers should carefully consider and interpret their own results in light of these findings.

## Figures and Tables

**Table 1:** Calculated electroactive surface of GCEs mechanically cleaned using different motion types.

Polishing Motion	8-Type			O-Type			I-Type		
Subseries	1	2	3	1	2	3	1	2	3
A [cm^2^]	0.1007 ± 0.0013	0.1010 ± 0.0016	0.1009 ± 0.0009	0.0942 ± 0.0053	0.0927 ± 0.0061	0.0894 ± 0.0068	0.0889 ± 0.0072	0.0915 ± 0.0033	0.0901 ± 0.0046
CV [%]	1.17	1.39	0.84	4.95	5.82	6.77	7.14	3.21	4.55
Ã [cm^2^]	0.1082 ± 0.0007			0.0921 ± 0.0033			0.0902 ± 0.0027		

**Table 2:** Technical parameters of the used potentiostats.

Parameter	Potentiostat				
	EmStat3	µStat 200	µAutolab Type III	Multi Autolab/M101	Autolab 302N
Maximum current [A]	±0.02	±2 × 10^−4^	±0.08 A	±0.1	±2–20 *
Potential range [V]	±3	±2	±5	±10	±30
Potential resolution [µV]	100	1000	3	3	0.3
Current range	1 nA–10 mA	1 nA–100 µA	10 nA–10 mA	10 nA–10 mA	10 nA–1 A
Number of current ranges	8	6	7	7	9
Price	lowest	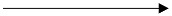			highest

* additional hardware module.

**Table 3:** Statistical parameters of the developed analytical procedures.

	Potentiostat				
	EmStat3	µStat 200	µAutolab Type III	Multi Autolab/M101	Autolab 302N
Linear range [mol/L]	1.0 × 10^−5^– 1.4 × 10^−4^	1.0 × 10^−5^– 8.0 × 10^−5^	1.0 × 10^−6^– 6.0 × 10^−5^	5.0 × 10^−6^– 1.0 × 10^−4^	4.0 × 10^−6^– 1.0 × 10^−4^
R^2^	0.99954	0.99936	0.99987	0.99897	0.99948
LOD^1^ [mol/L]	1.42 × 10^−8^	7.00 × 10^−9^	8.97 × 10^−9^	4.09 × 10^−9^	4.80 × 10^−9^
LOD^2^ [mol/L]	1.51 × 10^−8^	6.85 × 10^−9^	6.06 × 10^−9^	2.02 × 10^−9^	1.52 × 10^−9^
LOD^3^ [mol/L]	2.50 × 10^−9^	1.86 × 10^−6^	2.41 × 10^−7^	2.37 × 10^−8^	8.55 × 10^−7^
LOD^4^ [mol/L]	1.54 × 10^−6^	1.30 × 10^−6^	2.98 × 10^−7^	3.33 × 10^−7^	3.68 × 10^−7^

## Data Availability

The datasets used and/or analyzed during the current study are available from the corresponding author on reasonable request.
